# Editorial: Edible flowers: Understanding the effect of genotype, preharvest, and postharvest on quality, safety, and consumption

**DOI:** 10.3389/fpls.2022.1025196

**Published:** 2022-09-13

**Authors:** Valentina Scariot, Antonio Ferrante, Daniela Romano

**Affiliations:** ^1^Department of Agricultural, Forest and Food Sciences, University of Torino, Turin, Italy; ^2^Department of Agricultural and Environmental Sciences—Production, Landscape, Agroenergy, Università degli Studi di Milano, Milan, Italy; ^3^Department of Agriculture, Food and Environment, University of Catania, Catania, Italy

**Keywords:** phytochemicals, phenolics, antioxidant compounds, shelf-life, sensorial quality, health beneficial effect, anti-hyperglycemic properties, cold storage

## Biodiversity of edible flowers

The use of flowers as food is very ancient, since the times of the Greeks and Romans, when flowers were used to decorate various dishes and improve their aesthetic impact. They were part of the diet and improved the sensory quality of the dishes, thanks to their aromas, colors, texture, and taste. Edible flowers have always been widely used in the traditional cuisine in Asia and particularly in China, India, Thailand, and Japan. Today, the use of edible flowers is becoming more and more popular. Edible flowers can be used raw or fresh, as a garnish or an integral part of a dish. The flowers are consumed both as a conventional food and as a functional or nutraceutical food, thanks to the high and diversified content of bioactive compounds present in the various floral parts depending on the species (Falla et al., 2020).

The biodiversity of edible flowers is very wide. Alongside the commonly known edible flowers (e.g., *Rosa* hybr., *Chrysanthemum* × *morifolium* (Ramat.) Hemsl, *Dianthus caryophyllus* L., *Tagetes* L. spp., *Hemerocallis* L. spp., etc.), ornamental garden plants can be used (e.g., *Robinia pseudoacacia* L., *Acca sellowiana* (O. Berg) Burret, *Forsythia* × *intermedia* Zabel, *Bougainvillea glabra* Choisy) or wild species (e.g., *Glebionis segetum* Fourr., *Borago officinalis* L., *Malva sylvestris* L., *Centaurea cyanus* L., *Papaver rhoeas* L., etc.). In the list proposed by Lim (2014a,b) in the two books of the series dedicated to edible medicinal and non-medicinal plants relating to edible flowers, 1,729 species, belonging to 631 genera and 133 botanical families were listed. Of course, many of the lesser-known edible flowers are harvested in the wild and are not cultivated. Many species are found in natural or partially anthropized ecosystems, and sometimes edible wild flowers include traditional ecosystem weeds, such as *C. cyanus* or *Viola tricolor* L. (Benvenuti and Mazzoncini; Demasi et al., 2021a). For example, in the Indian context, edible flowers are widely used and constitute a significant part of the rural diet; Ray et al. found edible wild species belonging to 153 species and 59 botanical families.

Edible flowers, like any food product, must be primarily safe for the consumer. However, there are no clear legal rules such as the list of flowers allowed for consumption, growing conditions, harvest dates, storage conditions and substances authorized for the protection of edible flowers. The possibility of microbial contamination and the presence of chemical residues make it necessary to pay particular attention to the correct cultivation, harvesting, transport and storage of edible flowers. It is also necessary to raise awareness of this group of food products among those operating at different levels of the supply chain, as well as consumers. In the case of wild edible flowers, especially those that come from an area that is not in agricultural use, there may be a concern of chemical impurities absorbed from soil or from air (Matyjaszczyk and Smiechowska, 2019; Drava et al., 2020).

The possible allergenicity and toxicity of the flowers has to be taken in account (Lucarini et al., 2020). For instance, the Ranunculaceae family is rich in ornamental species but often toxic. Nevertheless, some genera (e.g. *Aquilegia, Caltha, Clematis*) are indicated for their edible flowers.

## Edible flowers and their potential benefit on human health

The introduction of flowers for consumption has raised questions about the value of edible flowers. Numerous researches have therefore investigated the composition of edible flowers, showing the richness of phytochemicals with bioactive properties, such as vitamins, minerals, and phenolics (Rop et al., 2012; Takahashi et al., 2020; Demasi et al., 2021a).

Most studies are focusing on phenolics, a wide group of non-nutritional plant secondary metabolites that possess several beneficial properties and exert a strong antioxidant activity, scavenging reactive oxygen species (Durazzo et al., 2019). Adequate intake of phenolics could confer benefits for human health, by reducing the risk of cardiovascular, dysmetabolic, and neurodegenerative diseases, and cancer (in particular gastrointestinal neoplasms), by playing anti-inflammatory effects, and by favorably modulating the gut microbiota composition (Devecchi et al., 2021).

Edible flowers rich in health protective phenolic compounds could therefore provide novel opportunities as ingredient and nutraceutical sources to combat the increasing prevalence of diet and lifestyle-influenced non-communicable chronic diseases (NCDs), such as type 2 diabetes (T2D) (Devecchi et al., 2021).

Among edible flowers, roselle (*Hibiscus sabdariffa* L.) red calyces - already consumed as part of traditional cuisines and processed foods in several countries of Asia and Africa—are rich in phenolic compounds, which potentially have high antioxidant and anti-hyperglycemic properties. *In vitro* assays of four different organic solvents (chloroform, hexane, ethyl acetate, and initial crude extraction in 100% methanol) extracted fractions of calyces of roselle indicated that calyces of roselle are excellent sources of health protective phenolic compounds with high antioxidant and anti-hyperglycemic functions and organic solvent (ethyl acetate and methanol) extracted fractions of this edible flower can be strategically utilized to design functional food ingredients and nutraceuticals (Banwo et al.).

However, the phenolic composition of edible flowers as well as the presence and concentration of other botanicals vary widely between different tissues, plant parts, different species and cultivars, and based on the growing condition and environment (Demasi et al., 2021a; Falla et al., 2021).

Additionally, phenolics can interact with other nutritional components leading to the formation of soluble or insoluble complexes that can affect overall bioavailability, bioaccessibility, and health relevant functional properties of phenolics and can alter the health protective functional qualities of plant foods and food ingredients (Banwo et al.). At present, the contribution of edible flowers to human metabolism *in vivo* is almost unexplored. A first human pilot study, analyzing the relationship between the dietary content of phenolics from edible roses and the urinary phenolic excretion in healthy volunteers, found a direct relationship between the increasing rose phenolic content and the phenolic excretion, meaning that phenolics have been absorbed and metabolized by the body (Devecchi et al., 2021).

Thus, *in vivo* and human studies are needed to define and confirm the potential role of edible flowers on human health.

## Postharvest handling and preservation of edible flowers

The postharvest chain of edible flowers must be carefully defined to ensure the highest quality to the consumer. Since the edible part is represented by the flower these must be characterized for their senescence process (Demasi et al., 2021b). Especially wild flowers are highly perishable, compared with the flowers from ornamental garden plants, because their stems are cut very short and they are usually dry stored (Toscano et al., 2021). Flowers are the most perishable organ of the plants. They are genetically programmed to be short-lived organ, in some flower the life span is limited to 1 day such as ephemeral flowers (*Hemerocallis, Hibiscus*, etc.). The metabolism of flowers is usually higher than other organs and characterized by higher respiration rates. Therefore, the primary strategy for preserving the edible flowers is to store and transport them at low temperature, such as below 5°C (Demasi et al., 2020). Low temperature reduces respiration and ethylene biosynthesis. Ethylene is a key regulator of flower senescence, especially in ethylene sensitive flowers (Reid and Wu, 1992). The preservation of the quality of edible flowers must be obtained by the identification of their ethylene production level and sensitivity to this plant hormone. Many flowers are very sensitive to ethylene, and they can senesce at concentration of 0.5 μl L^−1^. This concentration can be very often found in different step of the distribution chain or in the packaging. Flowers that are highly sensitive to ethylene must be protected from the ethylene action using specific inhibitor such as 1-methylciclopropene (1-MCP) or reducing its biosynthesis (Scariot et al., 2014).

As above describe the most used edible flowers are rich in antioxidant compounds that can help the flowers to counteract the senescence. Therefore, some of these antioxidant compounds can transiently increase and ensure a longer shelf life (Cavaiuolo et al., 2013).

In a study focused on four edible flower species, *Ageratum houstonianum* Mill*, Tagetes lemmonii* A. Gray*, Salvia dorisiana* Standl, and *Pelargonium odoratissimum* (L.) L'Hér ‘Lemon' showed different behavior during storage highlighting the different attitude of edible flowers to preserve the quality. These flowers stored at 4°C up to 6 days showed an increase of lipid peroxidation where the antioxidant capacity was retained in three species except *P. odoratissimum* (Marchioni et al.). This species also showed a significant reduction of carbohydrates during storage. Among the different edible flower species, *T. lemmonii is* very interesting because retains the total ASA concentration during storage and has a shelf-life.

## Conclusion

In conclusion, the biodiversity of edible flowers can become an important resource for gastronomic innovation, also for offering new flavors, colors (sensorial quality), and products with a high nutraceutical potential. Particularly, the wild edible flowers could be an important resource to obtain new food of interesting nutritional value; it is important, however, to investigate in view to better define which flowers can be used without problem on human health. Appropriate strategies of harvesting, cultivation, transport and storage must be used considering the postharvest physiology of edible flowers, their metabolism, composition in term of antioxidant compounds, ethylene production and sensitivity.

## Author contributions

All authors listed have made a substantial, direct, and intellectual contribution to the work and approved it for publication.

## Conflict of interest

The authors declare that the research was conducted in the absence of any commercial or financial relationships that could be construed as a potential conflict of interest.

## Publisher's note

All claims expressed in this article are solely those of the authors and do not necessarily represent those of their affiliated organizations, or those of the publisher, the editors and the reviewers. Any product that may be evaluated in this article, or claim that may be made by its manufacturer, is not guaranteed or endorsed by the publisher.
